# Risk Factors for Road Traffic Injury-Related Mortality in Iran; a Systematic Review and Meta-Analysis

**DOI:** 10.22037/aaem.v9i1.1329

**Published:** 2021-09-11

**Authors:** Mahmoud Yousefifard, Amirmohammad Toloui, Koohyar Ahmadzadeh, Mohammed I M Gubari, Arian Madani Neishaboori, Fatemeh Amraei, Saeed Safari, Alireza Baratloo, Vafa Rahimi-Movaghar, Mostafa Hosseini

**Affiliations:** 1 Physiology Research Center, Iran University of Medical Sciences, Tehran, Iran.; 2 Community Medicine, College of Medicine, University of Sulaimani, Sulaimani, Iraq.; 3 Emergency Medicine Specialist, Tehran University of Medical Sciences, Tehran, Iran.; 4 Emergency Department, Shohadaye Tajrish Hospital, Shahid Beheshti University of Medical Sciences, Tehran, Iran.; 5 Prehospital and Hospital Emergency Research Center, Tehran University of Medical Sciences, Tehran, Iran.; 6 Department of Emergency Medicine, Sina Hospital, Tehran University of Medical Sciences, Tehran, Iran.; 7 Sina Trauma and Surgery Research Center, Tehran University of Medical Sciences, Tehran, Iran.; 8 Brain and Spinal Injuries Research Center (BASIR), Neuroscience Institute, Imam Khomeini Hospital, Tehran University of Medical Sciences, Tehran, Iran.; 9 Department of Epidemiology and Biostatistics, School of Public Health, Tehran University of Medical Sciences, Tehran, Iran.

**Keywords:** Accidents, Traffic, Mortality, Wounds and Injuries, Risk factors

## Abstract

**Introduction:**

Gathering information regarding the risk factors of mortality and disability due to road traffic injuries can provide evidence for adopting effective interventions to reduce the burden of the injury. Therefore, the present study intends to identify the most important risk factors of road accident-related mortality in Iran by conducting a systematic review and meta-analysis.

**Methods:**

Search was done in English and Persian electronic databases, for articles published until the end of 2020. Cross-sectional, cohort and case-control studies were included. Risk factors were divided into age and sex, road related factors, exceeding speed limit, road user behaviors, vehicle related factors, weather condition, and light condition. Data were reported as adjusted odds ratio (OR) of death with 95% confidence interval (95% CI).

**Results:**

20 studies were included (2,682,434 traffic accident victims and 23,272 deaths; mortality rate=1.28%). The risk of death in road traffic injuries in men was 1.66 times higher than women (OR = 1.66; 95% CI: 1.03, 2.68) and with each year increase in age, the risk increased by 1% (OR = 0.01; 95% CI: 1.00, 1.01). In addition, accident in urban streets (OR = 1.76; 95% CI: 1.08, 2.88), roadway defects (OR = 2.15, 95% CI: 1.59, 2.91), and not driving on a flat and straight road (OR = 1.60; 95% CI: 1.14, 2.24) were the most important road-related risk factors for mortality. Exceeding the speed limit was another risk factor of death (OR = 3.16; 95% CI: 2.83, 3.54). However, regarding exceeding safe speed, only three studies have been included, which greatly reduces the power of analysis. Not maintaining focus on the road (OR = 2.99; 95% CI: 1.49, 6.04), not fastening seatbelt (OR = 3.11; 95% CI: 1.08, 8.91), and reckless overtaking (OR = 4.04; 95% CI: 3.34, 4.89) were independent road user-related risk factors for mortality. Risk of pedestrian mortality in comparison with drivers and passengers is 2.07 times higher (OR = 2.07; 95% CI: 1.53, 2.58). In addition, risk of death in accidents occurring during daylight hours (OR = 0.26; 95% CI: 0.18, 0.37) is lower than that of other hours. No significant relationship was present between mortality and vehicle types (four-wheeled vehicle: OR = 0.99; 95% CI: 0.050, 1.97; two-wheeled vehicle: OR = 0.75; 95% CI: 0.48, 1.16). In the case of vehicle-related factors, only 2 studies were included, which also dealt only with the type of vehicle (two-wheeled/four-wheeled). Vehicle-related factors such as the car model, its safety rating, and safety standards were not mentioned in any study.

**Conclusion:**

Low to very low-level evidence shows that there is a significant relationship between factors related to age, sex, road, road user, exceeding the speed limit, and light condition with the mortality of traffic accident victims. However, all studies included in the present study were retrospectively designed and the analyses were not adjusted for most of the key potential confounders. Therefore, it seems that despite years of effort by researchers in the field of traffic accidents in Iran, there is still no comprehensive and reliable picture of the most important risk factors for road accident mortalities in Iran.

## 1. Introduction:

Road traffic injuries are one of the most important causes of death worldwide, leading to more than 54 million new disabilities and 1.2 million deaths annually (1). World Health Organization report shows that 93% of road traffic injury mortalities occur in low- and middle-income countries, and these injuries are the leading cause of death in children and young adults, aged 5-29 years (2). In 1990, road accidents were the eighth most important reason for years of life lost, and were predicted to become the sixth leading cause of death and disability by 2017 (3). Road traffic injuries mostly involve young and middle-age groups. The rate of road accidents in developing countries is increasing, and its direct and indirect burdens are higher compared to developed countries (1). World Health Organization states that more research is needed on the epidemiological pattern of traffic accidents in low- and middle-income countries in order to determine the extent of the problem and identify vulnerable people in traffic accidents. There is no accurate estimate of the economic and social burden of traffic accidents in these countries, and Iran, as one of the low- and middle-income countries, is no exception (2).

Gathering information regarding the risk factors of mortality and disability due to road traffic injuries can provide evidence for adopting effective interventions for reducing the burden of the injury. Risk factors of road traffic injuries are generally divided into several subgroups such as road user-related factors, road-related factors, vehicle-related factors, environmental factors (light, temperature, humidity, weather conditions), and physiological factors (such as circadian rhythm) (4-10). 

Although valuable efforts have been made in recent years to identify risk factors for road traffic injury-related deaths in Iran (9, 10), the most important cause of road traffic injury-related mortality is not yet clear. In addition, no comprehensive study exists in this subject area, yet. Therefore, the present study intends to identify the most important risk factors of road accident-related mortality in Iran through a systematic review and meta-analysis.

## 2. Method

**2.1. Study design**

The present study was designed based on the “Meta-analysis of Observational Studies in Epidemiology” (MOOSE) guideline (14). The protocol of this study is not submitted in a registry. Moreover, the systematic review in the current study is performed based on the PRISMA guideline.

**2.2. Search strategy**

Two independent reviewers conducted extensive searches in electronic databases, including Medline, Embase, Scopus, and Web of Science to find articles published until the end of 2020. Also, Persian language databases including SID, Magiran, and CIVILICA were searched. The search strategy in four English language databases is reported in supplementary table 1.

Since systematic and advanced searches in Persian language databases are limited, different combinations of keywords related to road accidents, mortality and its risk factors were used in each database. To find additional articles or unpublished data, a manual search was performed in the list of relevant articles’ references. Google and Google scholar search engines were also searched, in both Persian and English languages.

**2.3. Selection Criteria**

In the present study, cross-sectional, cohort, and case-control studies investigating risk factors for road accident mortality in Iran were included. Exclusion criteria were the lack of result adjustment for key potential confounders, not reporting odds ratio (OR) or relative risk (RR), no reports regarding the approach for data collection, case-report studies, letter to the editor studies, failure to investigate risk factors for mortality, failure to investigate death as an outcome, case-series studies on deaths (absence of a living group), and review studies.

**2.4. Data Collection**

Search results were combined and duplicate studies were removed. Two independent researchers identified potentially eligible studies by reviewing and screening the titles and abstracts. Then, the full texts of these articles were studied, and finally, related articles were included. The same approach was adopted for Persian language databases. Finally, any disagreement was resolved by discussion with a third researcher.

The evaluated data in the present study included the name of the first author, publication year, geographical area of ​​the study (provincial or national), sample size, age and sex distribution, and risk factors reported in the articles. In cases where the findings were reported more than once, the study with the highest number of patients was included.

2.5. Data synthesis

The outcome of road accident was defined as mortality. Since a considerable diversity was observed in reported risk factors in the articles, researchers divided the articles into seven groups. Accordingly, risk factors were divided into age and sex, road-related factors, exceeding speed limit, road user behaviors, vehicle-related factors, weather condition, and light condition.

Most studies stratified their analyses based on road type including urban and out of city roads. In these cases, we entered the reports, separately. 

**2.6. Risk of bias assessment**

Quality control of the studies was performed according to the National Heart, Lung, and Blood institute (NHLBI) risk of bias assessment tool (11). This tool contains 14 questions about the quality of the study and the reported outcomes. The two researchers independently evaluated the quality of the articles, and based on their own judgment answered each question with: yes (low risk), no (high risk), cannot determine, not reported, or not applicable.

To report the overall risk of bias, based on our definition, three items were defined as fatal errors, including 1) assessment of exposure (risk factor) after examining the outcome (death), 2) failure to assess exposure and outcome clearly, validly, reliably, and consistently across all study participants and 3) failure to adjust the analysis for the most important potential key confounding factors. Therefore, articles that received no (high-risk), cannot be determined, or not reported answers in at least one of the questions in this category were labeled poor quality.

**2.7. The level of evidence**

Evidence level was assessed based on Grading of Recommendations, Assessment, Development and Evaluations (GRADE) criteria (12). According to the GRADE criteria, the level of evidence varies between High to very low. The score of observational studies starts from Low; therefore, if there was a risk of bias, imprecision, inconsistency, indirectness and publication bias, the level of evidence would be reduced by 1 to 2 points. If there was a large effect size, presence of dose-response and decrees in effect size after adjustment for confounders, one to two points were added to the quality of findings.

**2.8. Statistical analyses**

Data were entered into the statistical program and analyses were performed using STATA 17.0 software (Stata Corporation, College Station, TX). Data were reported as adjusted ORs of death and 95% confidence interval (95% CI). Heterogeneity between the studies was assessed using chi-square test and I^2^ statistics report, and p values less than 0.1 or I^2^ greater than 50% were considered significant (indicating heterogeneity). Since significant heterogeneity was expected to be observed between studies (due to differences in design and population), analyses were performed based on a random effect model. In all analyses, p <0.05 was considered significant. Egger’s test was used to identify publication bias (13).

## 3. Results

3.1. Characteristics of the imported articles

**Figure 1:**
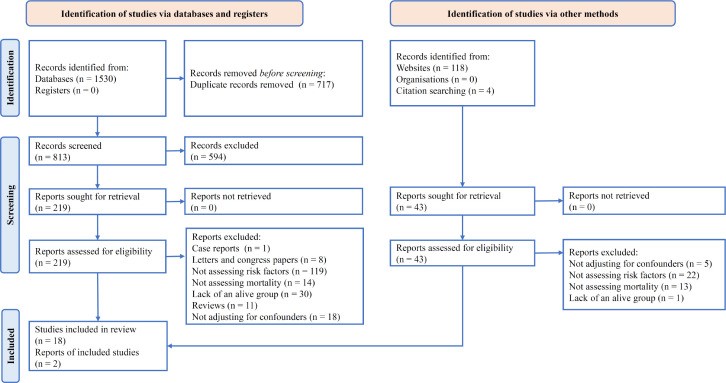
PRISMA flow diagram of the present study

A systematic search on Medline, Embase, Scopus and Web of Science resulted in 813 non-duplicate articles and after the initial screening, 219 articles were reviewed in more detail. Of these, 119 articles were excluded due to lack of studying risk factors, 13 articles due to not assessing mortality, 30 articles due to lack of a group of living casualties (no control group), and 18 articles due to the lack of adjustment for confounding factors in analyses, in addition to the 11 excluded review articles, eight excluded letters to the editor and congress abstract articles (lack of sufficient information), and one excluded case report article. Manual search in other sources yielded 122 non-duplicate articles and finally, 2 additional studies were included. Therefore, 20 original articles (14-33) were selected to assess the relationship between independent risk factors of mortality in road accidents in Iran (Figure 1).

There were 19 retrospective cross-sectional studies and one retrospective cohort study. Eight studies were based on national data and 12 studies were based on provincial or urban data to determine independent risk factors of Road traffic injury-related mortality. Four studies were performed only on accidents on urban roads, two studies on suburban or rural roads and 14 studies on data from both. These studies performed their analyses on a data collected between 2008 and 2018. These 20 studies included data on 2,682,434 traffic accident victims. With the exception of one study that did not report mortality, a summary of data from 19 studies showed that 23,272 deaths occurred among these accidents (1.28%). Table 1 shows a summary of the included articles.

**3.2. Risk factors assessed in the articles**

Risk factors related to road traffic injury-related mortality were classified into seven groups including road user-related factors, exceeding the speed limit, road-related factors, vehicle-related factors, weather condition, light condition, age, and sex.

Road-related factors included roadway defects, slippery road, the road not being flat and straight, urban road, suburban road, accident lane, and one-way/two-way road. The lane of the accident and one-way/two-way road factors were very different and could not be pooled in the analyses. Factors related to road user included not maintaining focus on the road, not fastening seatbelt, reckless overtaking, driver’s license, losing control of the vehicle, sudden lane excursion, fatigue, and helmet use. The only factor related to the vehicle was the type of vehicle, which was classified as four-wheel or two-wheel. Weather condition also included snowy or rainy, cloudy, or foggy, seasons, and air pollutants. Finally, light condition was divided into two groups: daylight hours (light hours) and night (dark hours). Table 2 reports the different risk factors studied, according to the number of reports of each of them in the analyses presented in the articles.

**3.3. Meta-analysis**

**The relationship between age and sex with road traffic injury-related mortality**

12 studies containing 16 separate analyses were included. These analyses included data from 1,601,183 injured patients. Pooled analysis showed that the odds of death in road traffic injuries in men is 1.66 times higher than women (OR = 1.66; 95% CI: 1.03, 2.68; p = 0.038).

**Figure 2:**
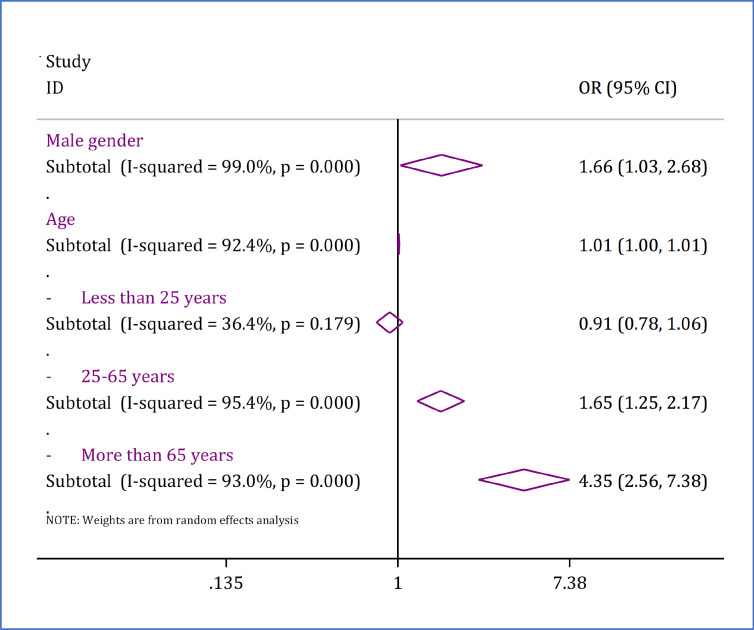
Forest plot for association of sex and age with road traffic injury-related mortality in Iran. OR: odds ratio; CI: confidence interval. Supplement table 2 shows details of individual studies in each subgroup

For examining the relationship between age and mortality due to road traffic injuries, two types of analyses were performed, since some studies included age as a continuous variable and some studies performed analyses according to age groups (categorical variable). It was found that with each year increase in age, the odds of death increased by 1% (OR = 0.01; 95% CI: 1.00, 1.01; p <0.0001). By examining the relationship between age groups and deaths due to road traffic injuries, it was found that the relationship between age and mortality is significant only in the ages of 25 to 65 years (OR = 1.65; 95% CI: 1.25, 2.17; p <0.0001) and over 65 years (OR = 4.35; 95% CI: 2.56, 7.38; p <0.0001) (Figure 2). Details of articles included in this section are reported in supplementary table 2.

**The relationship between road-related factors and mortality**

**Figure 3:**
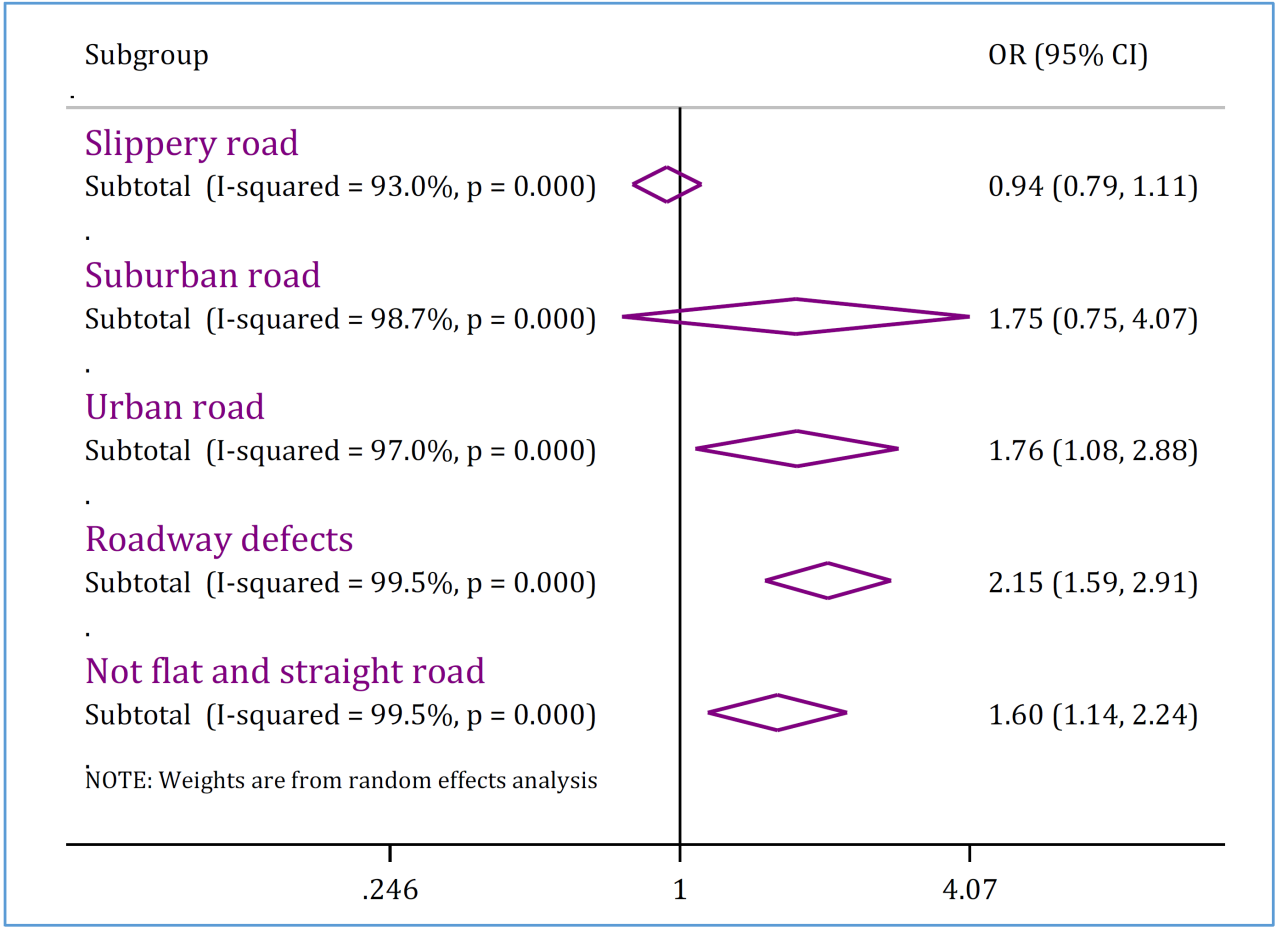
Road-related risk factors of mortality in traffic injuries in Iran. OR: Odds ratio; CI: Confidence interval. Supplement table 3 shows details of individual studies in each subgroup

In order to investigate the relationship between road-related risk factors and mortality, nine studies containing 94 separate analyses were included. These analyses included data on 1,958,574 traffic accident injuries. Pooled analysis showed that the odds of death in traffic accidents is higher on urban streets compared with suburban streets (interurban and rural roads) (OR = 1.76; 95% CI: 1.08, 2.88; p = 0.024). It was also found that roadway defects (OR = 2.15, 95% CI: 1.59, 2.91; p <0.0001) and not driving on a flat and straight road (OR = 1.60; 95% CI: 1.14, 2.24; p = 0.004) have a significant relationship with increase in the odds of mortality in traffic accidents (Figure 3). Details of articles included in this section are reported in supplementary table 3.

**The relationship between speeding and road accident mortality**

**Figure 4:**
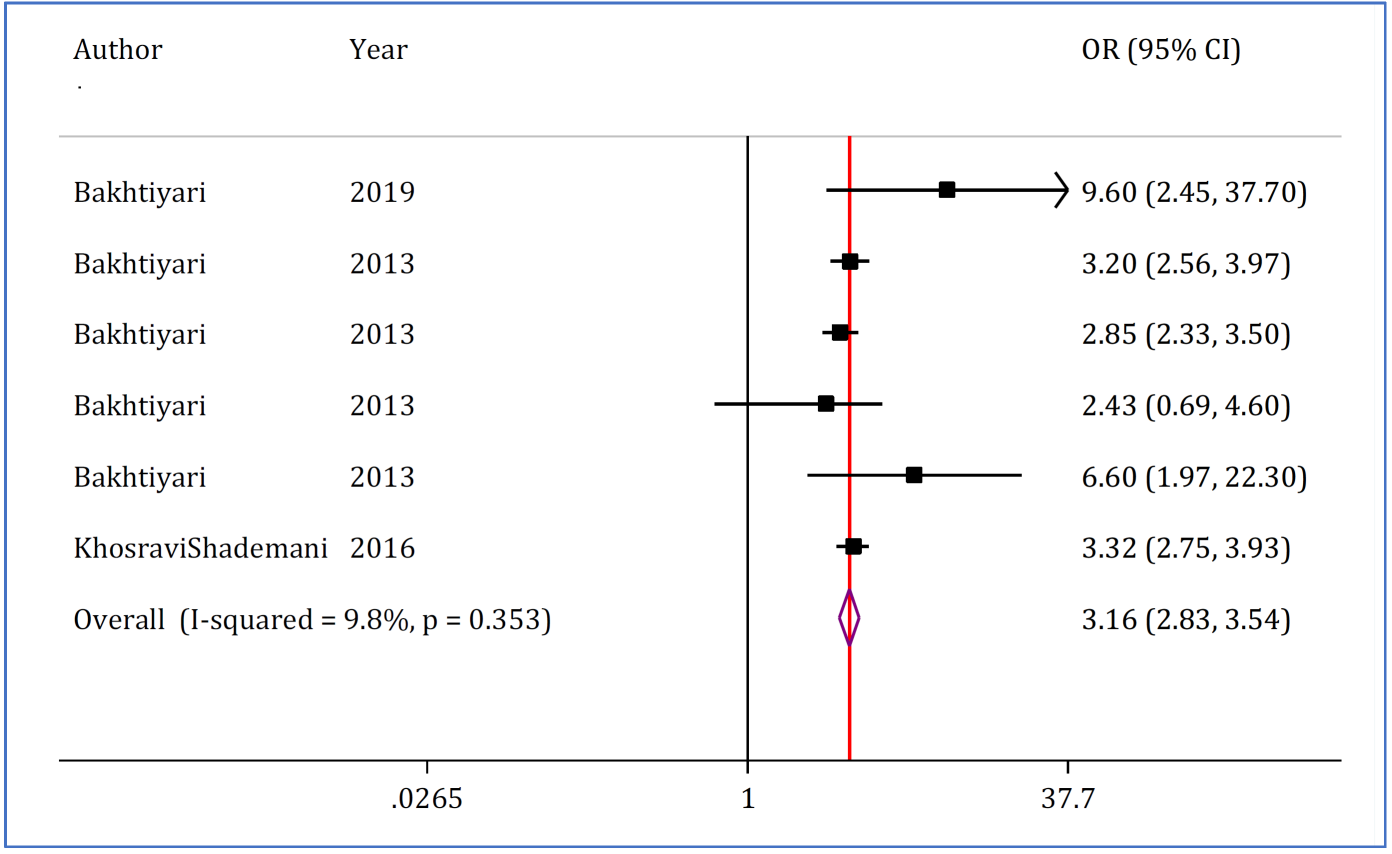
Forest plot of exceeding speed limit and mortality in traffic injuries in Iran. OR: Odds ratio; CI: Confidence interval

Only three studies containing six separate analyses examined the relationship between speeding and road accident mortality. These three studies included data on 664,291 traffic accident victims. Analyses in this section showed that exceeding the speed limit increases the odds of death in road accidents by up to 3.16 times (OR = 3.16; 95% CI: 2.83, 3.54; p <0.0001) (Figure 4).

**The relationship between road user factors and mortality**

**Figure 5:**
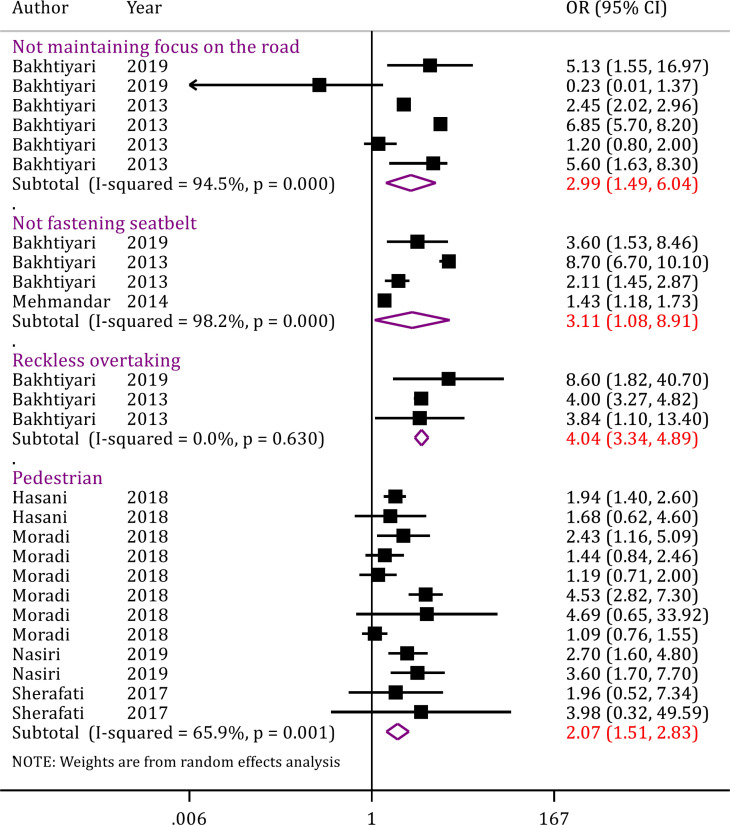
Road user-related risk factors of mortality in traffic injuries in Iran. OR: Odds ratio; CI: Confidence interval

Seven studies examined the relationship between road user-related factors and road accident mortality. These seven studies included 25 separate analyses containing data on 623,500 traffic accident injured patients. Analyses in this section showed that not maintaining focus on the road increases the odds of death in traffic accidents by 2.99 times (OR = 2.99; 95% CI: 1.49, 6.04; p = 0.002). Also, not fastening seatbelt and reckless overtaking increase the odds of death by 3.11 and 4.04 times, respectively (OR = 3.11; 95% CI: 1.08, 8.91; p = 0.035; OR = 4.04; 95% CI: 3.34, 4.89; p < 0.0001). It was also found that the risk of pedestrian mortality is 2.07 times higher in comparison with drivers and passengers (OR = 2.07; 95% CI: 1.53, 2.58; p <0.0001) (Figure 5).

**The relationship between vehicle-related factors and road accident mortality**

The only vehicle-related factor mentioned in the studies was the differences between four-wheeled and two-wheeled vehicles. Other factors such as car models or the cars’ manufacture dates were not reported in the studies.

**Figure 6:**
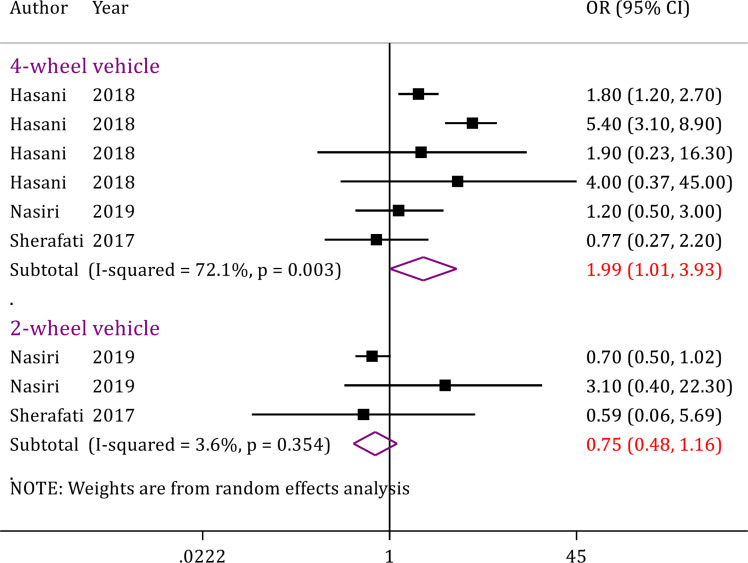
Vehicle-related risk factors of mortality in traffic injuries in Iran. OR: Odds ratio; CI: Confidence interval

In this section, only 3 studies (9 analyses) with a sample size of 21,182 patients were included. The interesting point in this section was that the mortality of traffic accidents in four-wheeled vehicles was significantly higher than that of two-wheeled vehicles (OR = 1.99; 95% CI: 1.01, 3.93; p = 0.048) (Figure 6). 

We performed a sensitivity analysis and excluded Hasani et al.’s study (18), since the study was performed on pedestrian mortality only. The findings revealed that no significant relationship was present between patient mortality and different vehicle types (Four-wheeled vehicle: OR = 0.99; 95% CI: 0.050, 1.97; p = 0.985; Two-wheeled vehicle: OR = 0.75; 95% CI: 0.48, 1.16; p = 0.198) (data not shown).

**The relationship between weather-related factors and road accident mortality**

In this section, included articles investigated weather conditions such as snowy/rainy, cloudy/foggy weather and different seasons. The analyses of this section included data of 1,577,998 road accident victims. Seven articles and 51 separate analyses were included in this section.

**Figure 7:**
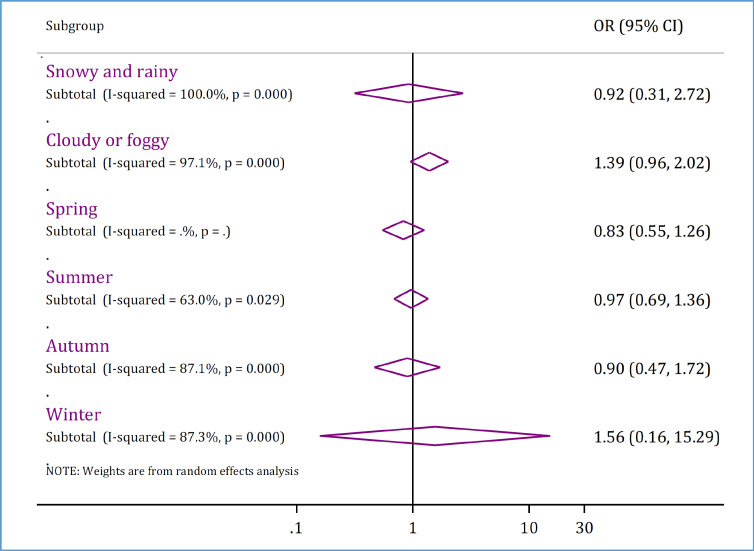
Weather-related risk factors of mortality in traffic injuries in Iran. OR: Odds ratio; CI: Confidence interval. Supplement table 4 shows details of individual studies in each subgroup

There was no relationship between weather conditions and road accident mortality. Pooled analysis showed that mortality from road accidents while driving on snowy or rainy roads (OR = 0.92; 95% CI: 0.31, 2.72; p = 0.996) and in cloudy or foggy weather (OR = 1.39; 95% CI: 0.96, 2.02; p = 0.085) is no different from sunny weather. Also, different seasons were not related to road accident mortality (Figure 7). Supplementary table 4 shows the details of the articles included in this section.

**Relationship between light condition and road accident mortality**

The study of light condition and its relationship with road accident mortality was performed in two parts of driving during daylight hours and dark hours of the day. Data of articles included in this section were divided into daytime, night, sunrise, and sunset. Since the level of light at sunrise and sunset is lower than that during the day, it was decided to include these times of day in the dark hour group.

**Figure 8:**
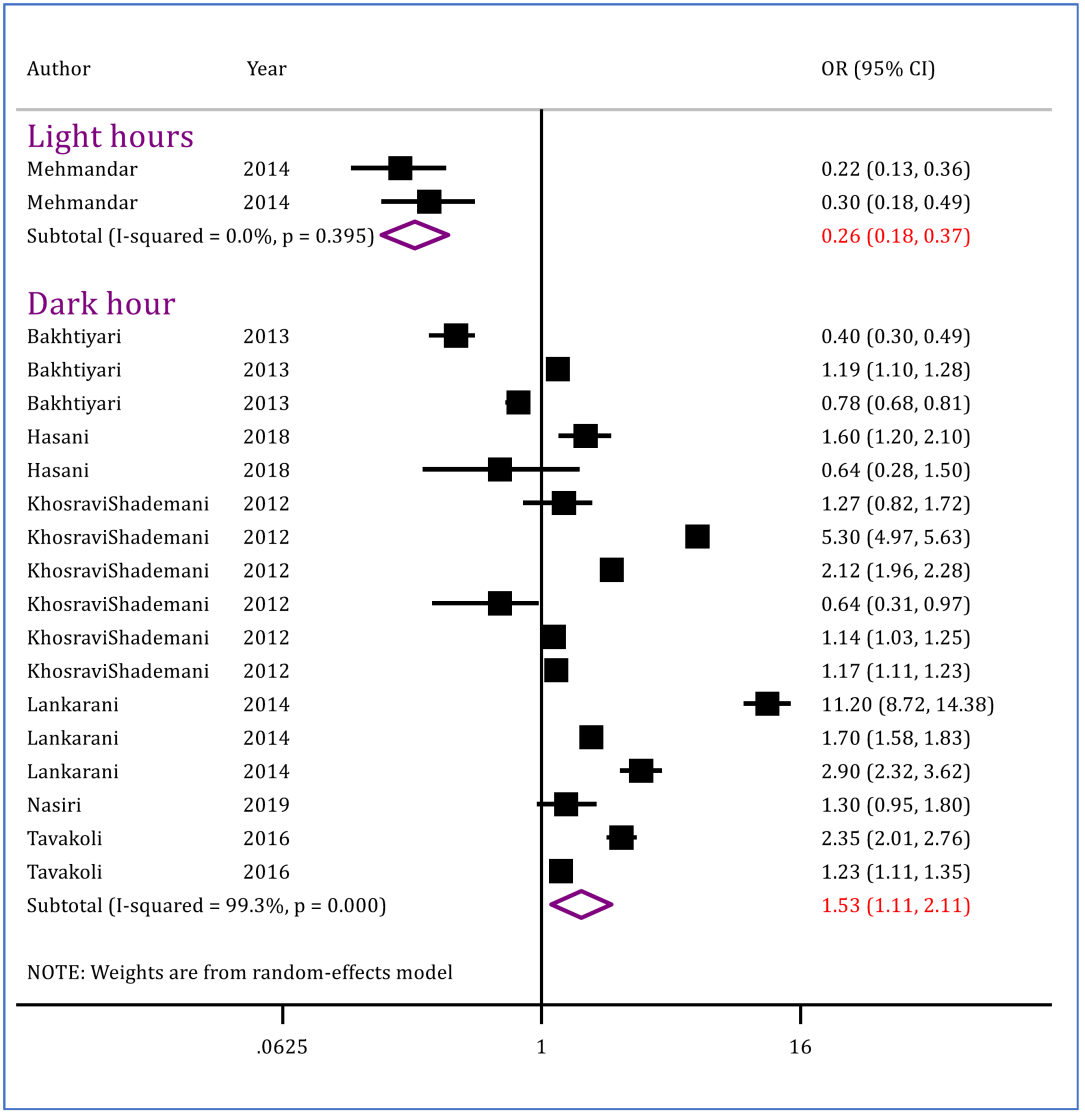
Light condition and risk of mortality in traffic injuries in Iran. OR: Odds ratio; CI: Confidence interval

In this section, data of seven articles (19 separate analyses) were entered, which included 2,145,907 traffic accident victims. Analyses showed that the odds of death in accidents occurring during daylight hours (OR = 0.26; 95% CI: 0.18, 0.37; p <0.0001) is lower than that of other hours. Accordingly, mortality in the dark hours of the day is higher than that of the light hours (OR = 1.53; 95% CI: 1.11, 2.11; p = 0.010) (Figure 8).

**Publication bias**

Egger’s test was used to check the publication bias. Analyses showed that there was evidence of bias in the age analysis section (p <0.0001). However, no publication bias was observed in the relationship between sex (p = 0.238), road-related factors (p = 0.510), speeding (p = 0.256), road user-related factors (p = 0.380), vehicle-related factors (p = 0.720), weather condition (p = 0.676), and light condition (p = 0.566) with mortality (supplementary figure 1).

**Risk of bias assessment**

Since all studies were performed retrospectively and no detailed information was provided on how data was collected in these articles, we were not able to determine whether the outcome was measured after the exposure or not. Therefore, all items in this section were labeled as cannot determine. None of the studies reported blinding status of the observer or data collector.

To examine item 15 of the NHLBI tool, regarding analysis adjustment for key potential confounders, the researchers in the present study decided to consider the articles as low-risk if they adjusted their analyses for road-related risk factors, road user-related risk factors, speeding of vehicle, and vehicle-related risk factors. Since none of the included studies had adjusted their analyses for all of these factors, based on our judgment, all articles were placed in the high-risk category.

Since the included studies had fatal errors in the two items of assessment of exposure (possible risk factors) before examining the outcome (occurrence of death) and adjusting the analyses for key potential confounding factors, overall risk of bias for all studies were considered high.

The level of evidence

Evidence level was assessed based on GRADE criteria, and accordingly, the score of observational studies starts from Low. As previously mentioned, there was a high risk of bias in all studies. Therefore, the level of evidence in all risk factors was rated down by one point. Also, in the assessment of relationship of mortality and sex, age, road-related, road user-related, and vehicle-related factors, weather condition, and light condition, serious inconsistency was observed in the analyses; therefore, the level of evidence was rated down at least one point. In the age analysis, evidence of publication bias was observed, which also reduces the level of evidence by 1 point. However, since all the analyses are multivariate models, and their effect size has decreased after adjusting for confounding factors, one point was added to the level of evidence in all sections. Finally, the level of evidence was low in exceeding the speed limit and very low in other factors (Table 4).

## 4. Discussion

The present study summarized the existing evidence in recognizing the risk factors of road accident mortality in Iran based on factors related to road, road user, speed, vehicle, and weather and light condition, for the first time. Low to very low-level evidence shows that there is a significant relationship between age, sex, road-related factors, road user-related factors, exceeding the speed limit, and light condition and mortality in traffic accidents. No correlation was found between weather conditions and vehicle-related factors and mortality.

All of the studies examined only a small fraction of the risk factors, and no study was found to fit at least one multivariate model with factors related to road, road user, speed, vehicle, weather and light condition. Therefore, any effect observed in the present study should be interpreted with caution. The researchers of the present study even searched the website of the Ministry of Health as well as research centers related to traffic accidents and trauma in the country to find a report that includes all possible risk factors for road accident mortality in the analysis. To the best of the researchers' knowledge, no such document or report exists.

The retrospective nature of the included studies is another major limitation of the findings. In retrospective studies, different people record information and it is not clear how accurate the data have been recorded. On the other hand, most of the articles did not mention whether the exposure assessment was performed before collecting the outcome data or not.

Regarding exceeding safe speed, only three studies have been included, which greatly reduces the power of analysis. Also, in the case of vehicle-related factors, only 2 studies were included, which also only dealt with the type of vehicle (two-wheeled/four-wheeled). Vehicle-related factors such as the car model, its safety rating, the presence of airbags and their number in the car, the existence of intelligent braking systems, balance maintenance, and other safety standards were not mentioned in any study.

Risk of bias assessment of the present study showed that there is a high risk of bias among studies. This is due to retrospective nature of studies, which raises concerns about the validity and the accuracy of findings. Therefore, it is suggested that more prospective studies be conducted in this field in the future.

**Table 1:** Summary of included studies

Study	Design	State / city	Road type	Sample size	Sampling year	No. male	No. mortality	Age	Category of risk factor	Independent risk factors for RTI-related mortality
Bakhtiyari, 2014 (15)	RCS	Iran	Urban / suburban	592168	2010	537688	210	34.1±14	Road user; speed; age; sex; light condition	Not maintaining focus on the road; exceeding the speed limit; not fastening seatbelt; light condition; reckless overtaking; age; sex
Bakhtiyari, 2019 (14)	RCS	Iran	Suburban	1160	2015	NR	37	NR	Speed; road user	Exceeding the speed limit; not maintaining focus on the road; not fastening seatbelt; reckless overtaking; not maintaining focus on the road
Dastoorpoor, 2016 (16)	RCS	Khuzestan	Urban	76006	2008-2015	73464	521	NR	Road; weather condition	Slippery road; snowy or rainy
Ghaem, 2017 (17)	RCS	Shiraz	NR	5840	2009-2014	3588	172	41.3±19.2	Age	Age
Hasani, 2018 (18)	RCS	Tehran / Alborz	Urban	10302	2013-2014	6466	407	37.16 ± 0.2	Road; road user; vehicle; weather condition; light condition; age; sex	Age; sex; type of vehicle (4-wheel); suburban road vs. urban road; season; cloudy or foggy; light condition; pedestrian
Hosseinpour, 2017 (19)	RCS	Isfahan	Urban / suburban	83648	2006-2010	74743	411	26.4 ± 14.3	Road; weather condition; age; sex	Age; sex; season; suburban road vs. urban road
Khorshidi, 2016 (20)	RCS	Iran	Urban / suburban	245326	2012-2013	NR	11087	NR	Road	Suburban road vs. urban road
Khosravi-Shademani, 2013 (29)	RCS	Iran	Urban / suburban	861074	2009	783577	NR	34.0 ± 10.6	Road; light condition; weather condition; sex; age	Roadway defects; slippery road; road not being flat and straight; light condition; cloudy or fogy; snowy; rainy; sex; age
Khosravi-Shademani, 2016 (27)	RCS	Iran	Suburban	70963	2012	69139	2744	0 to 99	Speed	Exceeding the speed limit
Lankarani, 2014 (21)	RCS	Iran	Urban / suburban	542863	2010	NR	3388	NR	Road; weather condition; light condition	Roadway defects; road not being flat and straight; slippery road; snowy or rainy; light condition
Mehmandar, 2014 (22)	RCS	Iran	Urban / suburban	2585	2008-2009	2558	805	NR	Road user; light condition; weather condition; age; sex	Not fastening seatbelt; season; light condition; age; sex
Moradi, 2018 (23)	RCS	Tehran	Urban	6405	2013-2014	4070	237	39.2 ± 19.5	Road user; age; sex	Pedestrian; age; sex
Mousazadeh, 2019 (24)	RCS	Tabriz	Urban	11238	2016-2018	7816	71	34.3 ± 16.2	Age	Age
Nasiri, 2019 (25)	RC	Kerman, Jiroft	Urban / suburban	8920	2011-2015	6850	143	NR	Road; road user; vehicle; light condition; age; sex	Urban road vs. suburban road; pedestrian; type of vehicle; light condition; age; sex
Paravar, 2014 (26)	RCS	Kashan	Urban / suburban	2000	2010-2011	1662	122	36.3 ± 20.8	Road; age; sex	Urban road vs. suburban road; age; sex
Sherafati, 2017 (28)	RCS	Langerod	Suburban	1520	2013-2014	1158	60	35.4±17.9	Road user; vehicle; weather condition	Pedestrian; type of vehicle; season; age;
Taravatmanesh, 2018 (30)	RCS	Rafsanjan	Urban / suburban	4899	2014-2015	3997	NR	28.1±15.8	Road; road user; light condition; age; sex	Urban road vs. suburban road; light condition; age; sex
Tavakoli, 2016 (31)	RCS	Iran	Urban / suburban	127995	2009-2012	NR	2161	NR	Road, light condition, age	Urban road vs. suburban road; light condition; age
Yadollahi, 2015 (32)	RCS	Shiraz	Urban / suburban	27222	2011-2014	18756	422	34 ± 15	Age; sex	Age; sex
Yousefzadeh, 2019 (33)	RCS	Rasht	Urban / suburban	300	2015	234	274	34.2 ± 19.1	Age	Age

NR: Not reported; RC: Retrospective cohort; RCS: Retrospective cross-sectional study; RTI: Road traffic injuries

**Table 2:** Reported risk factors of road traffic injury-related mortality in included studies

Risk factor	Number of analyses*
Age	49
Sex	16
Road-related factor	
Roadway defects	37
Slippery road	28
Road not being flat and straight	17
Urban road	10
Suburban road	7
Lane of accident	4
One-way/two-way road	4
Exceeding the speed limit	6
Road user-related factor	
Pedestrian	13
Not maintaining focus on the road	6
Not fastening seatbelt	4
Reckless overtaking	3
Driver’s license	2
Losing control of the vehicle	2
Sudden lane excursion	2
Fatigue	1
Helmet use	1
Vehicle-related	
4-wheel	8
2-wheel	3
Weather condition	
Snowy or rainy	28
Cloudy or foggy	7
Summer	5
Autumn	5
Spring	3
Winter	3
Dusty weather	1
NO	1
NO2	1
NOx	1
O3	1
PM10	1
Relative Humidity	1
SO2	1
CO	1
Sunny weather	1
Temperature	1
Total Evaporation	1
Windy weather	1
Light condition	
Light hours	14
Dark hour	9

*, In some cases, the number of analyses exceeded the number of articles because some studies reported findings based on different subgroups.

**Table 3:** The quality assessment of included papers regarding risk of bias

**Study**	**Q1**	**Q2**	**Q3**	**Q4**	**Q5**	**Q6**	**Q7**	**Q8**	**Q9**	**Q10**	**Q11**	**Q12**	**Q13**	**Q14**	**Overall**
Bakhtiyari, 2014	Yes	Yes	Yes	Yes	Yes	CD	Yes	NA	Yes	NA	Yes	NR	Yes	No	Poor
Bakhtiyari, 2019	Yes	Yes	Yes	Yes	No	CD	Yes	NA	Yes	NA	Yes	NR	Yes	No	Poor
Dastoorpoor, 2016	Yes	Yes	Yes	Yes	No	CD	Yes	NA	Yes	NA	Yes	NR	Yes	No	Poor
Ghaem, 2017	Yes	Yes	CD	Yes	No	CD	Yes	NA	Yes	NA	Yes	NR	Yes	No	Poor
Hasani, 2018	Yes	Yes	Yes	Yes	No	CD	Yes	NA	Yes	NA	Yes	NR	Yes	No	Poor
Hosseinpour, 2017	Yes	Yes	Yes	Yes	No	CD	Yes	NA	Yes	NA	Yes	NR	Yes	No	Poor
Khorshidi, 2016	Yes	Yes	Yes	Yes	No	CD	Yes	NA	Yes	NA	Yes	NR	Yes	No	Poor
Khosravi-Shademani, 2013	Yes	Yes	Yes	Yes	No	CD	Yes	NA	Yes	NA	Yes	NR	Yes	No	Poor
Khosravi-Shademani, 2016	Yes	Yes	Yes	Yes	No	CD	Yes	NA	Yes	NA	Yes	NR	Yes	No	Poor
Lankarani, 2014	Yes	Yes	Yes	Yes	No	CD	Yes	NA	Yes	NA	Yes	NR	Yes	No	Poor
Mehmandar, 2014	Yes	Yes	Yes	Yes	No	CD	Yes	NA	Yes	NA	Yes	NR	Yes	No	Poor
Moradi, 2018	Yes	Yes	Yes	Yes	No	CD	Yes	NA	Yes	NA	Yes	NR	Yes	No	Poor
Mousazadeh, 2019	Yes	Yes	Yes	Yes	No	CD	Yes	NA	Yes	NA	Yes	NR	Yes	No	Poor
Nasiri, 2019	Yes	Yes	CD	Yes	No	CD	Yes	NA	Yes	NA	Yes	NR	Yes	No	Poor
Paravar, 2014	Yes	Yes	CD	Yes	No	CD	Yes	NA	Yes	NA	Yes	NR	Yes	No	Poor
Sherafati, 2017	Yes	Yes	Yes	Yes	No	CD	Yes	NA	Yes	NA	Yes	NR	Yes	No	Poor
Taravatmanesh, 2018	Yes	Yes	CD	Yes	No	CD	Yes	NA	Yes	NA	Yes	NR	Yes	No	Poor
Tavakoli, 2016	Yes	Yes	Yes	Yes	No	CD	Yes	NA	Yes	NA	Yes	NR	Yes	No	Poor
Yadollahi, 2015	Yes	Yes	Yes	Yes	No	CD	Yes	NA	Yes	NA	Yes	NR	Yes	No	Poor
Yousefzadeh, 2019	Yes	Yes	CD	Yes	No	CD	Yes	NA	Yes	NA	Yes	NR	Yes	No	Poor

Yes: Low-risk; No: High-risk; CD: Cannot determined; NA: Not applicable; NR: Not reported

Questions: 1. Was the research question or objective in this paper clearly stated? / 2. Was the study population clearly specified and defined? 3. Was the participation rate of eligible persons at least 50%? / 4. Were all the subjects selected or recruited from the same or similar populations (including the same time period)? Were inclusion and exclusion criteria for being in the study prespecified and applied uniformly to all participants? / 5. Was a sample size justification, power description, or variance and effect estimates provided? / 6. For the analyses in this paper, were the exposure(s) of interest measured prior to the outcome(s) being measured? / 7. Was the timeframe sufficient so that one could reasonably expect to see an association between exposure and outcome if it existed? / 8. For exposures that can vary in amount or level, did the study examine different levels of the exposure as related to the outcome (e.g., categories of exposure, or exposure measured as continuous variable)? / 9. Were the exposure measures (independent variables) clearly defined, valid, reliable, and implemented consistently across all study participants? / 10. Was the exposure(s) assessed more than once over time? / 11. Were the outcome measures (dependent variables) clearly defined, valid, reliable, and implemented consistently across all study participants? / 12. Were the outcome assessors blinded to the exposure status of participants? / 13. Was loss to follow-up after baseline 20% or less? / 14. Were key potential confounding variables measured and adjusted statistically for their impact on the relationship between exposure(s) and outcome(s)?

**Table 4:** Assessment of level of evidence based on GRADE recommendation

Risk factor	Number of studies	Sample size	Risk of bias	Imprecision	Inconsistency (I^2^ range)	Indirectness	Publication bias	Judgment	Level of evidence
Sex	12	1601183	High	No serious imprecision	99.0%	No serious indirectness	Not present	Quality of evidence was rated down by 3 points since there is high risk of bias and very serious inconsistency. Evidence quality was rated up since the effect size was reduced by adjusting the analysis for confounders.	Very low
Age	13	1746996	High	No serious imprecision	36.4% to 95.4%	No serious indirectness	Likely	Quality of evidence was rated down by 3 points since there is high risk of bias, serious inconsistency, and possible publication bias. Evidence quality was rated up since the effect size was reduced by adjusting the analysis for confounders.	Very low
Road related	9	1958574	High	No serious imprecision	93.0% to 99.5%	No serious indirectness	Not present	Quality of evidence was rated down by 3 points since there is high risk of bias and very serious inconsistency. Evidence quality was rated up since the effect size was reduced by adjusting the analysis for confounders.	Very low
Speed	3	664291	High	No serious imprecision	9.8%	No serious indirectness	Not present	Quality of evidence was rated down by 1 point since there is high risk of bias. Evidence quality was rated up since the effect size reduced by adjusting the analysis for confounders.	Low
Road user related	7	623500	High	No serious imprecision	0.0% 98.2%	No serious indirectness	Not present	Quality of evidence was rated down by 3 points since there is high risk of bias and very serious inconsistency. Evidence quality was rated up since the effect size was reduced by adjusting the analysis for confounders.	Very low
Vehicle	2	21182	High	Serious	3.6% and 72.1%	No serious indirectness	Not present	Quality of evidence was rated down by 3 points since there is high risk of bias, serious imprecision, and serious inconsistency. Evidence quality was rated up since the effect size was reduced by adjusting the analysis for confounders.	Very low
Weather condition	7	1577998	High	No serious imprecision	63.0% 100%	No serious indirectness	Not present	Quality of evidence was rated down by 3 points since there is high risk of bias and very serious inconsistency. Evidence quality was rated up since the effect size was reduced by adjusting the analysis for confounders.	Very low
Light condition	7	2145907	High	No serious imprecision	99.3%	No serious indirectness	Not present	Quality of evidence was rated down by 3 points since there is high risk of bias and very serious inconsistency. Evidence quality was rated up since the effect size was reduced by adjusting the analysis for confounders.	Very low

## 5. Conclusion

Low to very low-level evidence shows that there is a significant relationship between factors related to age, sex, road, road user, exceeding the speed limit, and light condition with the mortality of traffic accident victims. However, all studies included in the present study had a retrospective design and the analyses were not adjusted for most of the key potential confounders. In addition, regarding exceeding safe speed, only three studies have been included, which greatly reduces the power of analysis. Also, in the case of vehicle-related factors, only 2 studies were included, which also dealt only with the type of vehicle (two-wheeled/four-wheeled). Vehicle-related factors such as the car model, its safety rating, the presence of airbags and their number in the car, the existence of intelligent braking systems, balance maintenance, and other safety standards were not mentioned in any study. Therefore, it seems that despite years of effort by researchers in the field of traffic accidents in Iran, there is still no comprehensive and reliable picture of the most important risk factors for road accidents mortalities in Iran.

## 6. Declarations:

### 6.1. Acknowledgment

None.

### 6.2. Conflict of interest

There is no conflict of interest. 

### 6.3. Authors’ contribution

**Study design:** MH, MY, VR; **Data gathering:** MY, AT, KA, MIMG, AMN, FA; **Analysis:** MH, MY; **Interpretation of results:** SS, AB, VR; **Drafting:** MY, AT, AMN; **Revising:** All authors

### 6.4. Funding

This study was funded and supported by Sina Trauma and Surgery Research Center, Tehran University of Medical Sciences (TUMS); (Grant no. 96-01-38-34527).
